# Potential Therapeutic Effects of NAMPT-Mediated NAD Biosynthesis in Depression In Vivo

**DOI:** 10.3390/brainsci12121699

**Published:** 2022-12-11

**Authors:** Jue Wang, Runxuan Sun, Linhan Xia, Xinying Zhu, Qi Zhang, Yilu Ye

**Affiliations:** 1 School of Basic Medical Sciences and Forensic Medicine, Hangzhou Medical College, Hangzhou 310053, China; 2 School of Clinical Medicine, Hangzhou Medical College, Hangzhou 310053, China; 3 School of Medical Imaging, Hangzhou Medical College, Hangzhou 310053, China; 4 College of Medicine, Jiaxing University, Jiaxing 314001, China

**Keywords:** nicotinamide adenine dinucleotide (NAD), depression, *Nampt*^flox/flox^ mice, chronic unpredictable mild stress (CUMS), nicotinamide phosphoribosyltransferase (NAMPT)

## Abstract

This study aimed to investigate the potential therapeutic effects of nicotinamide phosphoribosyltransferase (NAMPT)-mediated adenine dinucleotide (NAD) biosynthesis in depression models in vivo. *Nampt*^flox/flox^ mice were used to evaluate the role of NAMPT in depression. NAMPT and NAD levels in the prefrontal cortex (PFC) were measured, and depression-associated behavior, cognitive function, and social interaction were evaluated. The expression levels of BDNF, pCREB, CREB, monoamine neurotransmitters, and corticosterone (CORT) were also detected in the PFC. The contents of NAMPT and NAD decreased in the PFC in *Nampt*^flox/flox^ mice. *Nampt*^flox/flox^ mice showed depression-like behaviors, cognitive function deterioration, decreased social ability, and decreased dominance. Meanwhile, there were decreased expression levels of the pCREB/CREB ratio, but not BDNF, in the PFC. Levels of DA, 5-HT, and NE were decreased, and CORT was activated in the PFC of *Nampt*^flox/flox^ mice. Additionally, the role of NAMPT-NAD was examined in rats treated with nicotinamide riboside (NR) after being exposed to chronic unexpected mild stress (CUMS). NR reversed the decreased NAMPT expression in the PFC and HIP, and the NAD content in the PFC, but not HIP in rats with CUMS-induced depression. NR also improved depressive- and anxiolytic-like behaviors, locomotor activity, and cognitive function. BDNF expression and the pCREB/CREB ratio were significantly increased in both the PFC and HIP after NR treatment. The activation of CORT and decreased content of DA were reversed after NR treatment in the PFC. There was no difference in the 5-HT content among groups in both the PFC and HIP. Taken together, NAD synthesis induced by NAMPT could be associated with depression-like behaviors in mice, and the elevated NAD level by NR improved depression in rats.

## 1. Introduction

Approximately 4.4% of the worldwide population suffers from depression, a prevalent mental disorder [1]. However, uncertainty persists regarding the mechanisms underlying the etiology of depression and its therapeutic targets. Clinically, most classic antidepressants target the monoamine neurotransmitter system. However, these agents exhibit side effects, have safety concerns and residual symptoms, and prompt drug tolerance [2], all factors which need to be overcome. Owing to the limited efficacy of depression treatments, it is necessary to investigate new targets and treatment strategies.

The involvement of the prefrontal cortex (PFC) in depression has been previously recognized [3]. Recent studies have begun to explain how the PFC and its circuitry contribute to the symptoms of anxiety and depression. Therefore, the PFC is a significant site for the study of depression [4].

Numerous studies have demonstrated that synaptic plasticity is impaired by changes in neurotransmission, immune system malfunction, metabolic dysfunction, and other factors that collectively cause various functional abnormalities, including a depressed mood [5,6,7]. Lower NAD levels were linked to metabolic and neurodegenerative diseases, etc. In this regard, increasing or restoring intracellular NAD concentrations through NAD precursors, including nicotinamide (NAM) or nicotinamide riboside (NR), and enhancing enzyme activity has proved to be successful [8]. NR, a precursor of NAD, is a micronutrient that promotes energy metabolism and has neuroprotective effects. In depressed patients, energy metabolism is often disturbed in the brain, and NR is a relatively promising therapeutic agent.

Nicotinamide phosphoribosyltransferase (NAMPT) is necessary for the recycling of NAM to NAD, which is part of the salvage pathway of NAD synthesis [9]. Mammals have three NAD synthetase routes, with the salvage pathway being the most significant [10]. NR is the primary metabolite in the NAD salvage pathway whose level can also influence that of NAD, and thus the corresponding signaling pathway. They are all capable of directly influencing NAD levels, which are essential for maintaining cell cycle, metabolism, and cellular senescence [11]. Meanwhile, NAD plays a critical role in many depression-related signaling pathways. For instance, NAD is able to suppress depression by increasing SIRT1 activity. Therefore, numerous neurodegenerative disorders may exhibit decreased NAD levels due to decreased NAMPT expression or activity [12].

However, whether and how NAMPT-mediated NAD biosynthesis affects depression in vivo remains unclear. To obtain a higher specificity and exclude other confounding factors, we chose to use genetic knockout mice, *Nampt*^flox/flox^ mice, to investigate its role in behavior. Moreover, the PFC function plays an important role in the development of depression. Therefore, we selected the PFC as the site for viral injection. Meanwhile, NR, one of the NAD precursors, was employed to treat the depression model in rats with chronic unpredictable mild stress (CUMS). The therapeutic effects of NR were evaluated using depression-like behavior tests, neurotransmitters, and NAMPT-NAD expression. Hence in this study, *Nampt*^flox/flox^ mice and rats with a depression model induced by CUMS were treated with NR to explore the role of NAMPT-mediated NAD biosynthesis in depression in vivo.

## 2. Materials and Methods

### 2.1. Animals

Under 1% pentobarbital sodium anesthesia (40 mg/kg, i.p.) adeno-associated viruses with the Cre-recombinant enzyme and green fluorescent protein (GFP) gene (Cre–GFP) were stereotactically injected into the mouse PFC (Original point: Fontanel; AP: +1.9 mm; L: −0.4 mm; R: +0.4 mm; V: 2.2 mm) using a stereoscopic positioner, as presented by Dr. WeiPing Zhang (Zhejiang University). Mice with no green fluorescence in the PFC were excluded after four weeks. Male SD rats weighed 160–180 g at the time of purchase. All animals had free access to water and food in air-conditioned room (20~26 °C, relative humidity 40~50%) on a 12-h light/dark cycle. The Animal Health, Ethics and Research of Hangzhou Medical College approved the study procedures.

### 2.2. Treatment Groups

The mice were assigned to three groups: wild type (*Nampt*^wt/wt^), *Nampt*^flox/wt^ (*Nampt*^fl/wt^), and *Nampt*^flox/flox^ (*Nampt*^fl/fl^). After four weeks from Cre–GFP injection, several behavioral and other detections were conducted to evaluate changes in the mice.

The rats were divided into four groups: normal (normal), depression (model), sertraline (sertraline), and NR (NR). Normal and model rats received water devoid of NR, while rats in the NR group were given water containing 12 mM NR as needed. Gavage was used to administer sertraline (10 mg/kg). Dosing with NR or sertraline was continued for 50 days, including the day of the behavioral tests. The water bottles were changed twice a week.

### 2.3. Chronic Unpredictable Mild Stress (CUMS)

**Figure 1:**
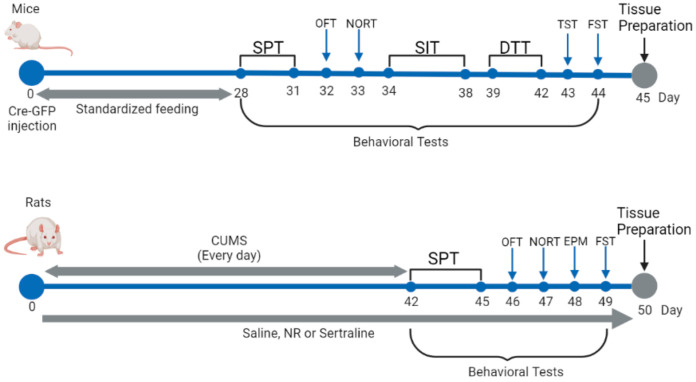
Schedules of the experimental protocol.

A previously published protocol has proven to be useful for inducing CUMS [13]. The stresses included food deprivation for 8 h, water deprivation for 8 h, inversion of the day/night cycle, forced swimming for 3 min, a 45° tilted cage for 8 h, soiled cage bedding for 8 h, and an empty bottle for 8 h. Except for the normal group, each rat in the rest of the groups was exposed randomly to one modest stressor every day following the NR treatment, which lasted for 42 days. Subsequently, the rats were evaluated with a series of behavioral assays. The schedules of the experimental protocol can be seen in Figure 1.

### 2.4. Sucrose Preference Test (SPT)

This test was developed in an earlier study evaluating anhedonia [14]. First, the animals were acclimated to two water bottles. Pure water was placed in one bottle, whereas a 1% sucrose solution was placed in the other. The animals were initially acclimatized, evaluated for two more days, and then tested for 3 h. The positions of the bottles were switched throughout the testing period. The percentages of sucrose and total liquid consumed were calculated. The less the sucrose solution was consumed, the more severe the depression-like behavior of the animal became.

### 2.5. Open Field Test (OFT)

Individual animals were gently positioned in the central region of a well-lit, open area. The spontaneous activity system continually recorded and evaluated locomotor paths for five minutes using the VisuTrack system (Xinruan Information Technology Co., Ltd., Shanghai, China). The total distance and velocity of travel, as well as the time and distance spent in the central region, were documented.

### 2.6. Elevated Plus Maze (EPM)

A labyrinth was used to situate creatures across an open arm. Five minutes of unrestricted maze exploration was allowed, and the total distances traveled and the distance ratios, together with the total time taken, were recorded and calculated. Tracks were recorded and analyzed using a video analyzer (Ethovision XT, Noldus, Netherlands).

### 2.7. Novel Object Recognition Test (NORT)

Animals were placed in the empty chamber during habituation to explore freely for five minutes. In T_1_ phase, they were placed into their home cages for five minutes, during which two identical objects (familiar object: cubic crude wood with 4 cm diameter) were placed in the chamber oppositely. After a 1.5-h time interval, animals were put into the chamber again for five minutes, when one of the familiar objects was replaced with a novel one (novel object: cylindrical crude wood with 4 cm diameter and height); that was the T_2_ phase. Tracks were recorded, and the exploration time and exploration number were analyzed using the VisuTrack system. The computational formula was presented in a study by Ye et al. [13] to calculate the discrimination ratio (DR) and discrimination index (DI). A 1.5-cm window was used to define exploration as the distance between the animal’s snout and the object in front of it.

### 2.8. Dominance Tube Test (DTT)

Utilizing the tube test, social dominance was evaluated [15]. A plexiglass tube (length, 30 cm; inner diameter, 3 cm) with two small acrylic boxes (10 cm^3^) was attached to the end. The mice were trained for three days to move through the tube. In the test, two mice with equivalent body weights were positioned at the tube ends and were allowed to move into the tube and meet at the center. The “winner” of the trial was the animal that ejected the other from the tube, with the winner scoring 2 and the loser scoring 0. If there was no winner or loser, each mouse received a score of 1. The trials lasted for a maximum of 2 min.

### 2.9. Social Interaction Test (SIT)

A SIT was conducted in three chambers as previously described [16]. In the first phase, each mouse was placed into a chamber where there was no other mouse to adapt to the environment for 10 min. In the second phase, a familiar mouse in a cage was placed on the left chamber, and an empty cage was placed on the opposite chamber. In the third phase, an unfamiliar mouse in a cage was placed on the right chamber. Finally, the time that the mice stayed in each chamber was recorded, and the social preference index (SPI) (time stayed with the strange mice in the second phase/total time × 100) and social novelty index (SNI) (time stayed with the strange mice in the third phase/total time × 100) were calculated. The duration of time that the mice stayed in each chamber was recorded with a video camera and analyzed using the VisuTrack system.

### 2.10. Tail Suspension Test (TST)

To create an inescapable, oppressive environment, the tails of the mice were fixed, with their heads facing down in a wooden box (55 cm height × 15 cm width × 11.5 cm depth). The animals tried desperately to escape yet were unable to do so in this environment. The test was measured for 6 min, with the last 4 min recorded with a video camera to evaluate the immobility time, which was analyzed using the VisuTrack system.

### 2.11. Forced Swimming Test (FST)

Each animal was placed into a transparent glass tank (mouse: 60 cm height, 25 cm diameter; rat: 80 cm height, 40 cm diameter) with water at 25 ± 1 °C to adapt to the environment for 15 min. Immobility time was recorded when the animal stopped struggling in the water and was floating with only slight limb movements to keep its head above the water. The next day, the animals were subjected to swimming for 6 min, and the last 4 min were recorded to evaluate the immobility time, which was analyzed using the VisuTrack system.

### 2.12. BDNF, CREB, pCREB, and NAMPT Expression in the PFC and HIP

The PFC and HIP regions of the brain were immediately placed on ice after dissection. After sonication, total protein was extracted and quantified with a kit. Next, 60 μg of total protein was used for electrophoretic analysis for 2 h and transferred to a PVDF membrane at 100 V for 2 h under cooling conditions. pCREB/CREB (1:5000, Cell Signaling Technology), NAMPT (1:500, Abcam), and the membranes were first treated with GAPDH (1:10,000) overnight, followed by incubation with the secondary antibody conjugated to HRP (KangChen Bio-tech, Shanghai, China). Finally, the signal intensity was evaluated with Image J.

### 2.13. Detection of NAD, CORT, DA, 5-HT, and NE Levels

Tissue homogenates were centrifuged at 2000× *g* at 4 °C, and specialized ELISA kits were used to measure the contents of NAD, CORT, DA, 5-HT, and NE (Shanghai Elisa Biotech Co., Ltd., China). Optical densities were measured using a plate reader, and concentrations were calculated against standard curves.

### 2.14. Statistical Methods

Statistical significance was set at *p* < 0.05. The results are shown as the mean and SEM. Except for the effects on the NORT and DTT, differences were analyzed through a repeated-measures Bonferroni post hoc test and one-way ANOVA. The NORT was evaluated using a *t*-test to compare differences in DR in the NORT. The DTT was evaluated using non-parametric analysis with a Mann–Whitney test to compare the difference in winning points.

## 3. Results

### 3.1. NAMPT-NAD-CREB Expression in Nampt^flox/flox^ Mice and Behavioral Changes

#### 3.1.1. NAMPT-NAD Expression Is Decreased in PFC in Nampt^flox/flox^ Mice

**Figure 2:**
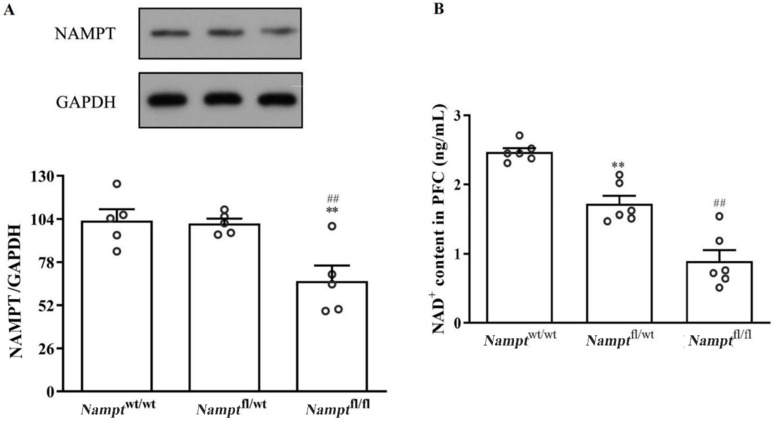
Reduced levels of NAMPT-NAD in the PFC of *Nampt*^flox/flox^ mice. (**A**) Levels of NAMPT in PFC. (**B**) Levels of NAD in PFC. ** *p* < 0.01 vs. *Nampt*^wt/wt^, ^##^ *p* < 0.01 vs. *Nampt*^flox/wt^.

NAMPT expression in *Nampt*^flox/flox^ mice was reduced to 64.67% compared to that in *Nampt*^wt/wt^ mice (Figure 2A, F(2, 12) = 8.963, *p* = 0.003). However, *Nampt*^flox/wt^ mice showed similar expression to that of *Nampt*^wt/wt^ mice (Figure 2A, F(2, 12) = 8.963, *p* = 0.869). NAMPT expression was also reduced in the PFC of *Nampt*^flox/flox^ mice relative to that in the PFC of *Nampt*^flox/wt^ mice (Figure 2A, F(2, 12) = 8.963, *p* = 0.004).

The NAD content was detected though ELISA. Compared with the *Nampt*^wt/wt^ group, the NAD content in the PFC was significantly lower in both the *Nampt*^flox/wt^ (Figure 2B, F(2, 15) = 44.134, *p* = 0.001) and *Nampt*^flox/flox^ mice (Figure 2B, F(2, 15) = 44.134, *p* = 0.000), and was further decreased in *Nampt*^flox/flox^ mice relative to *Nampt*^flox/wt^ mice (Figure 2B, F(2, 15) = 44.134, *p* = 0.001).

#### 3.1.2. Reduction in Locomotor Activity and Depression-Like Behavior in Nampt^flox/flox^ Mice

**Figure 3:**
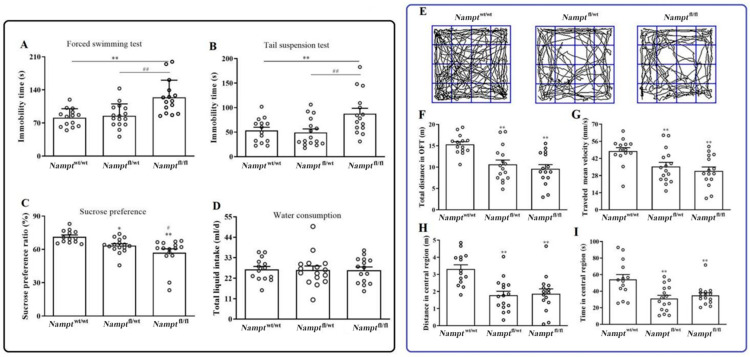
Behavioral assessment using FST, TST, SPT, and OFT in *Nampt*^flox/flox^ mice. (**A**) Immobility in FST. (**B**) Immobility in TST. (**C**) Sucrose preference ratios in SPT. (**D**) Total liquid intakes in SPT. (**E**) Typical tracks in OFT. (**F**) Total distances covered in OFT. (**G**) Mean velocities in OFT. (**H**) Distances in the center. (**I**) Total time spent in the center. * *p* < 0.05, ** *p* < 0.01 vs. *Nampt*^wt/wt^ mice; ^#^ *p* < 0.05, ^##^ *p* < 0.01 vs. *Nampt*^fl/wt^ mice.

The progress of depressive-like behaviors was examined using the FST (Figure 3A), TST (Figure 3B), and SPT (Figure 3C,D). The results suggested that immobility time in the FST (Figure 3A vs. *Nampt*^wt/wt^, F(2, 42) = 16.163, *p* = 0.001; vs. *Nampt*^flox/wt^, F(2, 42) = 15.623, *p* = 0.002), and TST (Figure 3B, F(2, 42) = 6.163, vs. *Nampt*^wt/wt^, *p* = 0.000; vs. *Nampt*^flox/wt^, *p* = 0.000) both increased when compared to *Nampt*^wt/wt^ and *Nampt*^flox/wt^ mice, which manifested despair-state formation in *Nampt*^flox/flox^ mice. In the SPT, the sucrose preference ratio decreased in the *Nampt*^flox/wt^ (Figure 3C, F(2, 42) = 10.325, *p* = 0.011) and *Nampt*^flox/flox^ mice (Figure 3C, F(2, 42) = 10.325, vs. *Nampt*^wt/wt^, *p* = 0.000; vs. *Nampt*^flox/wt^, *p* = 0.049). Total liquid intake was identical in all groups (Figure 3D, F(2, 42) = 0.012, *p* = 0.125).

Four weeks after the intraventricular injection, the mice were tested using the OFT. This resulted in a reduced total distance (Figure 3F, F(2, 42)=10.879, *p* = 0.000), travel velocity (Figure 3G, F(2, 42) = 6.682, *p* = 0.003), central distance (Figure 3H, F(2, 42) = 10.762, *p* < 0.001), and central time (Figure 3I, F(2, 42) = 7.818, *p* = 0.011) for *Nampt*^flox/flox^ mice compared to *Nampt*^wt/wt^ mice. The decrease in central distance and central time manifested as decreased explorative behaviors. Similar changes were observed in *Nampt*^flox/wt^ mice. However, the *Nampt*^flox/flox^ and *Nampt*^flox/wt^ groups did not differ in explorative behaviors.

#### 3.1.3. Reduction in Social Behaviors, Cognitive Function, and Social Dominant Position in Nampt^flox/flox^ Mice

**Figure 4:**
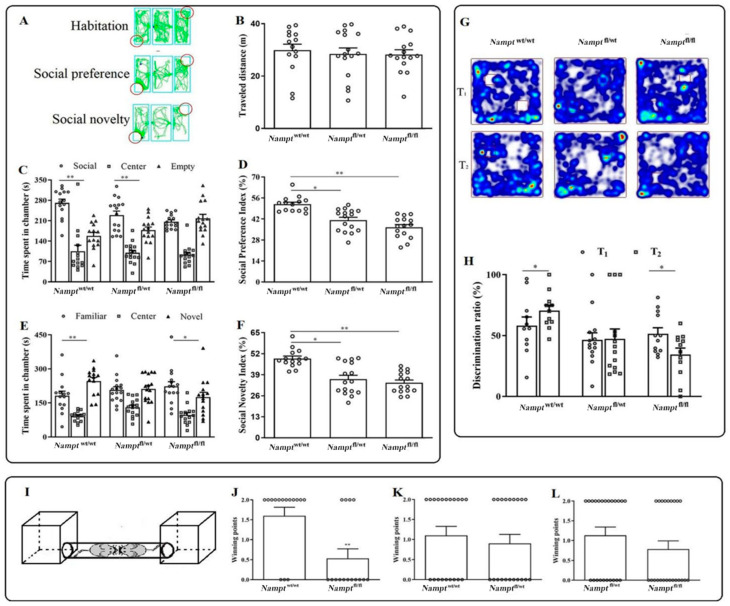
Behaviors in the SIT, NORT, and DTT in *Nampt*^flox/flox^ mice. (**A**) Schematic diagram of the three-chambered test with an explanation of the three 10-min sessions. (**B**) Traveled distance during the habituation session. (**C**) Time spent during the sociability session in three chambers. (**D**) SPT during the sociability session. (**E**) Time spent during the sociability session in the three chambers. (**F**) SNI during the social novelty session. (**G**) Heatmap of NORT. (**H**) DR in NORT. (**I**) Schema of the DTT explaining the procedure of the test session. (**J**) Wining points in groups between *Nampt*^wt/wt^ and *Nampt*^flox/flox^ mice. (**K**) Wining points in groups between *Nampt*^wt/wt^ and *Nampt*^flox/wt^ mice. (**L**) Wining points in groups between *Nampt*^flox/wt^ and *Nampt*^flox/flox^ mice. ** *p* < 0.01 vs. social chamber in Figure 4C, vs. familiar chamber in Figure 4E, with * *p* < 0.05, ** *p* < 0.01 vs. *Nampt*^wt/wt^ mice in Figure 4D, Figure 3F. ** *p* < 0.01 vs. *Nampt*^wt/wt^ mice in Figure 4J and ** *p* < 0.01 vs. T_1_ phase in Figure 3H.

Three successive sessions of the sociability test lasting 10 min each were conducted using three-chambered boxes (Figure 4A). The overall distance traveled was essentially the same in all the groups (Figure 4B, F(2, 42) = 0.144, *p* > 0.05). During the sociability session, *Nampt*^flox/flox^ (Figure 4C, F(2, 42) = 28.760, *p* = 0.000) and *Nampt*^flox/wt^ animals (Figure 4C, F(2, 42) = 13.481, *p* = 0.003) remained longer in the social chamber, although no differences were observed in the *Nampt*^flox/flox^ mice (Figure 4C, F(2, 39)=0.195, *p* = 0.571). Similar to the time spent in the chambers, the SPI decreased in both the *Nampt*^flox/wt^ (Figure 4D, F(2, 42)=19.726, *p* = 0.000) and *Nampt*^flox/flox^ animals (Figure 4D, *p* = 0.000), but not in *Nampt*^wt/wt^ animals. During the social novelty session, *Nampt*^wt/wt^ mice stayed longer in the novel chamber (Figure 4E, F(2, 42) = 26.242, *p* = 0.000). The time spent in the novel and familiar chambers did not differ significantly in *Nampt*^flox/wt^ mice (Figure 4E, F(2, 42) = 11.195, *p* = 0.071). However, *Nampt*^floxflox^ mice preferred the familiar compartment and stayed longer than in the novel compartment (Figure 4E, F(2, 42) = 22.737, *p* = 0.003). The SNI across the 10-min test period was significantly lower in the *Nampt*^flox/wt^ (Figure 4F, F(2, 42) = 16.571, *p* = 0.000) and *Nampt*^flox/flox^ animals (Figure 4F, F(2, 42) = 16.571, *p* = 0.000). This indicates that *Nampt*^flox/wt^ or *Nampt*^flox/flox^ mice had impaired sociability and social novelty preference.

In the NORT, DR increased in the T_2_ phase in *Nampt*^wt/wt^ mice compared with that in the T_1_ phase (Figure 4H, F(1, 20) = 0.035, *p* = 0.034). However, T_1_ and T_2_ were similar in the *Nampt*^flox/wt^ mice (Figure 4H, F(1, 26) = 2.240, *p* = 0.939). In contrast, the DR in the T_2_ phase in *Nampt*^flox/flox^ mice was lower than that in the T_1_ phase (Figure 4H, F(1, 22) = 0.007, *p* = 0.027).

In Figure 4I, a dominance tube test schematic depicting two mice competing in a cylindrical tube with a 30-mm diameter is shown. The number of victories for each mouse was counted, averaged, and represented using a histogram to determine the winning points. The winning points of *Nampt*^wt/wt^ mice were higher than those of *Nampt*^flox/flox^ mice (Figure 4J, *p* = 0.0092, non-parametric analysis), whereas no difference was observed between the *Nampt*^wt/wt^ and *Nampt*^flox/wt^ mice (Figure 4K, *p* = 0.752), or the *Nampt*^flox/wt^ and *Nampt*^flox/flox^ mice (Figure 4L, *p* = 0.376).

#### 3.1.4. Levels of BDNF-CREB and Monoamine Neurotransmitters Changed in the Prefrontal Cortex in Nampt^flox/flox^ Mice

**Figure 5:**
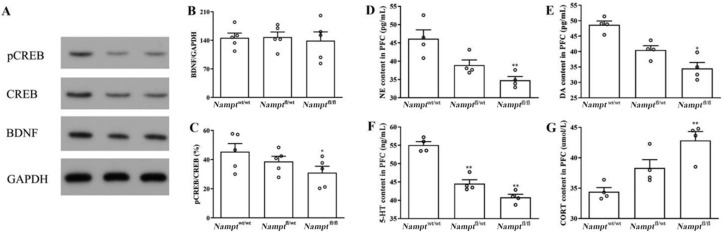
BDNF, CREB, NE, DA, 5-HT, and CORT levels in the PFCs of *Nampt*^flox/flox^ mice. (A) Band of WB. (**B**) BDNF expression in PFC. (**C**) pCREB/CREB expression in PFC. (**D**) NE content in PFC. (**E**) DA content in PFC. (**F**) 5-HT content in PFC. (**G**) CORT content in PFC. * *p* < 0.05, ** *p* < 0.01 vs. *Nampt*^wt/wt.^ mice.

BDNF levels in the PFC were similar in all the groups (Figure 5B, F(2, 12) = 0.085, *p* = 0.25). The pCREB/CREB expression decreased in the PFC of *Nampt*^flox/flox^ mice compared to that in the PFC of *Nampt*^wt/wt^ mice (Figure 5C, F(2, 12) = 2.453, *p* = 0.047).

As with the contents of monoamine neurotransmitters, the contents of NE and DA in the PFC decreased in *Nampt*^flox/flox^ mice (Figure 5D, *p* = 0.004; Figure 5E, *p* = 0.015) but not in *Nampt*^flox/wt^ mice (Figure 5D, F(2, 9) = 10.866, *p* = 0.051; Figure 5E, F(2, 9) = 6.888, *p* = 0.185) relative to the *Nampt*^wt/wt^ animals. However, the 5-HT content in the PFC decreased in both *Nampt*^flox/wt^ mice (Figure 5F, F(2, 9) = 16.940, *p* = 0.008) and *Nampt*^flox/flox^ mice (Figure 5F, *p* = 0.001). Relative to *Nampt*^wt/wt^ animals, the CORT content increased in *Nampt*^flox/flox^ mice (Figure 5G, *p* = 0.003); however, there was no difference in the *Nampt*^flox/wt^ animals (Figure 5G, F(2, 9) = 11.704, *p* = 0.171).

### 3.2. Effects of NR on Depression in Rats

#### 3.2.1. Levels of NAMPT and NAD in PFC and HIP with NR Treatment in CUMS-induced Depression Rats

**Figure 6:**
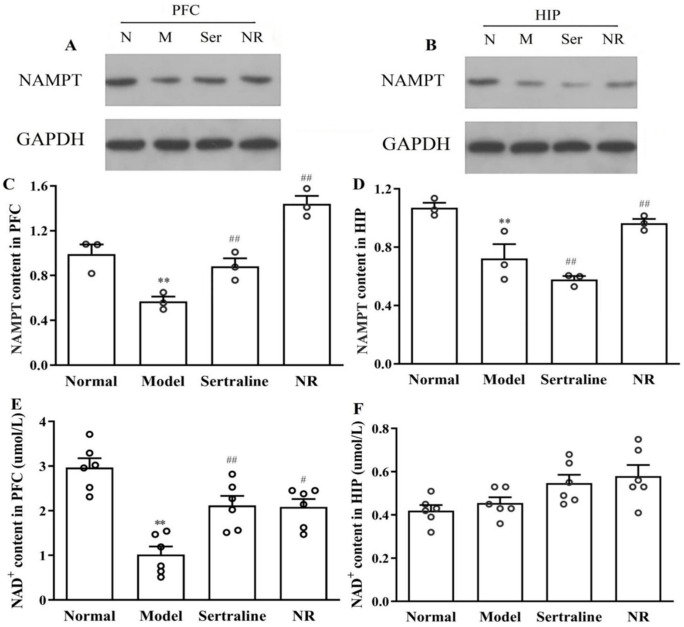
NAMPT expression as well as the NAD content in the PFC and HIP in rats with CUMS-induced depression with NR treatment. (**A**, **C**) NAMPT in the PFC. (**B**, **D**) NAMPT in the HIP. (**E**) NAD in the PFC. (**F**) NAD in the HIP. ** *p* < 0.01 vs. the normal group; # *p* < 0.05, ## *p* < 0.01vs. the model group.

Western blot analysis revealed that NAMPT expression in the PFC (Figure 6A, Figure 5C, F(3, 32) = 104.04, *p* = 0.000) and HIP (Figure 6B, Figure 5D, F(3, 32) = 65.649, *p* = 0.000) was significantly decreased after CUMS compared to that in normal animals. NR treatment reversed the CUMS-induced decrease in NAMPT expression in the PFC (Figure 6A, Figure 5C, *p* = 0.000) and HIP (Figure 6B, Figure 5D, *p* = 0.000) in animals, especially in the PFC. Sertraline also increased NAMPT expression in the PFC (Figure 6A, Figure 5C, *p* = 0.000) but decreased NAMPT expression in the HIP group (Figure 6B, Figure 5D, *p* = 0.005).

NAD decreased to 43.59% (Figure 5E, F(3, 20) = 16.669, *p* = 0.000) in the PFC of rats with CUMS-induced depression. NR (Figure 6E, *p* = 0.006) and sertraline (Figure 6E, *p* = 0.004) treatment increased the NAD content in the PFC. The HIP NAD content did not differ between the different groups (Figure 6F).

#### 3.2.2. Amelioration of Depression- and Anxiety-Associated Behavior with NR Treatment in CUMS-Induced Depression Rats

**Figure 7:**
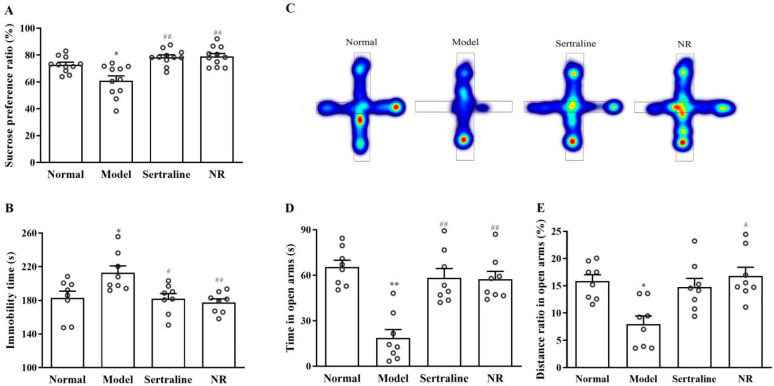
Amelioration of depression- and anxiety-associated behavior with NR treatment in rats. (**A**) Ratio of preferred sucrose in SPT. (**B**) Time spent in FST without moving. (**C**) Heatmap in EPM. (**D**) Spending time in open arms in EPM. (E) EPM distance ratio in open arms. * *p* < 0.05, ** *p* < 0.01 vs. the normal group; ^#^ *p* < 0.05, ^##^ *p* < 0.01 vs. the model group.

The sucrose preference ratio decreased by 15.27% in the model relative to that in the normal rats (Figure 7A, F(2, 28) = 2.826, *p* = 0.040). The sertraline and NR treatments reversed the sucrose preference ratio, reaching 80.07% (Figure 7A, *p* = 0.009) and 80.60% (Figure 7A, *p* = 0.006), respectively. In the FST, relative to the normal rats, the time spent immobile was longer in the CUMS group (Figure 7B, F(2, 28) = 5.535, *p* = 0.018). Treatment with sertraline (Figure 7B, *p* = 0.022) and NR (Figure 7B, *p* = 0.007) reversed the increase in immobility time after CUMS.

Relative to normal animals, the time (Figure 7D, F(2, 28)=6.779, *p* = 0.002) and distance ratio (Figure 7E, F(2, 28) = 4.557, *p* = 0.036) in open arms decreased in the EPM of rats. NR treatment reversed the time (Figure 7D, *p* = 0.002) and distance ratio (Figure 6E, *p* = 0.015) in open arms. Sertraline treatment reversed this effect (Figure 7D, *p* = 0.009) but not the distance ratio (Figure 7E, *p* = 0.095) in open arms.

#### 3.2.3. Improvement of Locomotor Activity and Cognitive Function with NR Treatment in CUMS-Induced Depression Rats

**Figure 8:**
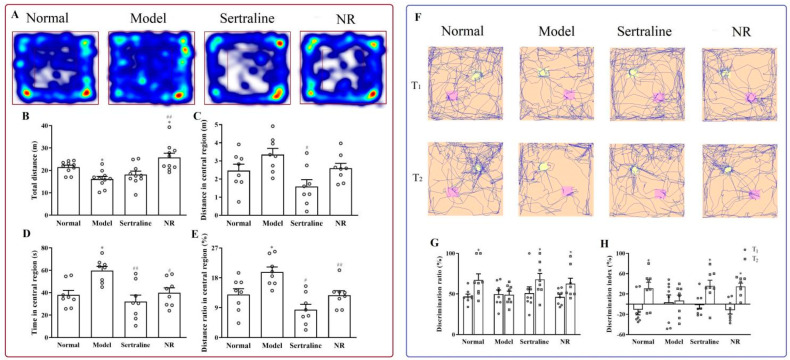
Improvement of locomotor activity and the cognitive function with NR treatment in rats. (**A**) Heatmap and typical tracks in OFT. (**B**) Distance traveled overall in OFT. (**C**) Distance covered in the central region. (**D**) Time spent in the center region. (**E**) Distance ratio in the center region. (**F**) Typical tracks in NORT. (**G**) DR in NORT. (**H**) DI in NORT. * *p* < 0.05 vs normal group; ^#^ *p* < 0.05, ^##^ *p* < 0.01 vs. model group in Figure 8B−E. Data in Figure 8G,H were analyzed using *t*-tests. * *p* < 0.05 vs. T_1_ in DR (Figure 8G) and in DI (Figure 8H).

In the OFT, the total distance decreased (Figure 8B, F(3, 28) = 7.559, *p* = 0.030), whereas both the time and distance ratios in the central region were extended (Figure 8D, F(3, 28)=7.016, *p* = 0.002; Figure 8E, F(3, 28) = 8.935, *p* = 0.005) after CUMS. NR treatment increased the total distance relative to the CUMS model animals (Figure 8B, *p* = 0.000). The total distance was greater than that in the normal control group after NR treatment (Figure 8B, *p* = 0.035). The NR and sertraline treatments reversed the time and distance ratios in the central region (Figure 8D, *p* = 0.005; Figure 8E, *p* = 0.004). However, there was no difference in the distance in the central region after NR treatment (Figure 8C, F(3, 28) = 4.584, *p* = 0.125), and this distance decreased with sertraline treatment (Figure 8C, *p* = 0.001).

In the T_1_ phase of the NORT, there were two objects with identical shapes. The time spent on each object was equal, which was manifested through a similar DR value (Figure 8F). In the T_2_ phase, both DI and DR were increased in the normal group (Figure 8G, F(1,14) = 4.817, *p* = 0.034; Figure 8H, F(1,14) = 0.016, *p* = 0.025), sertraline group (Figure 8G, F(1,14) = 3.682, *p* = 0.035; Figure 8H, F(1,14) = 2.024, *p* = 0.033), and NR group (Figure 8G, F(1,14) = 2.719, *p* = 0.048; Figure 8H, F(1,14) = 0.011, *p* = 0.011) over that in the T_1_ phase. However, no differences were observed in the model rats (Figure 8G, F(1,14) = 2.874, *p* = 0.941; Figure 8H, F(1,14)=0.850, *p* = 0.873).

#### 3.2.4. Levels of BDNF, pCREB/CREB, CORT, DA, and 5-HT in the PFC or HIP with NR Treatment in CUMS-Induced Depression Rats

**Figure 9:**
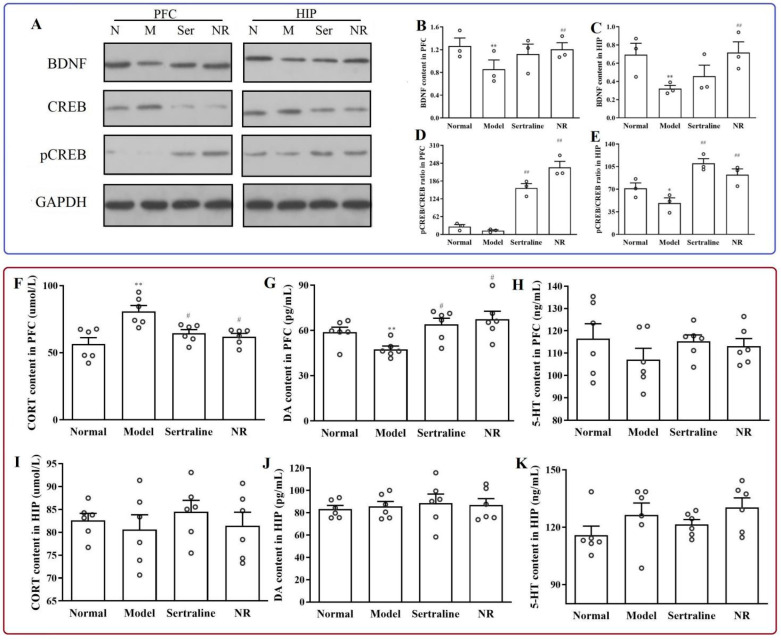
Levels of BDNF, pCREB/CREB, CORT, DA, and 5-HT in the PFC and HIP with NR treatment in CUMS-induced depression rats. (**A**) Levels of BDNF and TrkB in the PFC and HIP. (**B**) Levels of BDNF in the PFC. (**C**) BDNF levels in the HIP. (**D**) pCREB/CREB ratio in the PFC. (**E**) pCREB/CREB ratio in the HIP. (F) CORT level in the PFC. (**G**) DA level in the PFC. (**H**) 5-HT level in the PFC. (**I**) CORT content in the HIP. (**J**) DA content in the HIP. (**K**) 5-HT content in the HIP. * *p* < 0.05, ** *p* < 0.01 vs. normal group; ^#^ *p* < 0.05, ^##^ *p* < 0.01 vs. model group.

BDNF expression in the PFC (Figure 9B, F(3, 32) = 5.789, *p* = 0.001) and HIP (Figure 9C, F(3, 32) = 12.775, *p* = 0.000) decreased in rats with CUMS-induced depression. NR treatment reversed BDNF expression in the PFC (Figure 9B, *p* = 0.002) and HIP (Figure 9C, *p* = 0.000). Sertraline treatment did not alter BDNF levels in the PFC (Figure 9B, *p* = 0.109) and HIP (Figure 9C, *p* = 0.077). There was little pCREB/CREB expression in the normal and model groups in the PFC (Figure 9D, F(3, 32) = 217.616, *p* = 1.000). Compared to the normal group, pCREB/CREB expression decreased in the HIP (Figure 9E, F(3, 32) = 40.314, *p* = 0.000) in rats with CUMS-induced depression. NR significantly increased pCREB/CREB expression in the PFC (Figure 9D, *p* = 0.000) and HIP (Figure 9E, *p* = 0.000).

After CUMS, the CORT content increased (Figure 9F, F(3, 20) = 8.136, *p* = 0.001), and DA content decreased (Figure 9G, F(3, 20) = 5.803, *p* = 0.007) in the PFC. NR and sertraline reversed the CORT content (Figure 9F, F(3, 20) = 8.136, *p* = 0.010; Figure 9F, F(3, 20) = 8.136, *p* = 0.030, respectively) and DA content (Figure 9G, F(3, 20) = 8.136, *p* = 0.010; Figure 9G, F(3, 20) = 8.136, *p* = 0.040, respectively) in the PFC. However, there was no significant difference in the CORT (Figure 9I, F(3, 20) = 0.412, *p* = 1.000) and DA (Figure 9J, F(3, 20) = 0.155, *p* = 1.000) content between the groups in the HIP. There were no differences in 5-HT levels among the groups in rats with CUMS-induced depression (Figure 9H,K).

## 4. Discussion

We utilized *Nampt*^flox/flox^ mice and long-term treatment with NR in rats with CUMS-induced depression and found that NAMPT-mediated NAD synthesis played a pivotal role in depression. They both led to significant changes in depression-like behaviors, locomotor activity, social behaviors, cognitive function, and social dominance positions. In addition, the levels of NAMPT, NAD, BDNF, pCREB/CREB, monoamine neurotransmitters, and CORT were changed in *Nampt*^flox/flox^ mice and rats with CUMS-induced depression.

Numerous researchers have focused on the PFC and hippocampus in their efforts to explore the brain function underlying depression since these regions show structural and functional alterations that may be caused by altered brain circuits and neurotransmitters [17]. We targeted the PFC, where NAMPT was inhibited in this study. It was demonstrated that approximately 40% of NAMPT expression, followed by approximately 60% of NAD content, was downregulated. The hippocampus and PFC have been investigated in relation to depression [18]. After NR treatment, the expression of NAMPT in the hippocampus and PFC, as well as NAD in the PFC, all increased, except NAD in the hippocampus, which contributed to the improvement of behavioral deterioration in depression. The different NAD levels between the PFC and hippocampus may be related to their different functions.

NAD functions as an enzyme cofactor in various cellular activities [19] and is a vital component of SIRT1 signaling. The NAD-dependent deacetylase SIRT1 has been linked to depression [20]. Recent studies [21] have shown that NAD, a SIRT1-related pathway, is crucial for controlling emotion-related behaviors. Because of the enhancing mitochondrial activity and bioproduction of NAD, it played a significant role in brain aging and neurodegenerative diseases through reducing damaged mitochondria in models that accelerate premature aging [22].

Three processes can be adopted to create NAD [23]. Mammals most often use the salvage pathway, which starts with NAM [24]. NAMPT catalyzes the first step, producing nicotinamide mononucleotides through a combination of NAM with 5-phosphoribosyl pyrophosphate (NMN). This is followed by the adenylation of NMN to generate NAD. However, NAM is expensive and demonstrates low bioavailability. The NR biosynthetic pathway is distinctly and incredibly effective in rodents and humans [25]. Knock-out of NAMPT in the PFC caused significant depression-like behaviors, locomotor deterioration, and cognitive and social impairment, which was consistent with the results in rats with CUMS-induced depression. NR treatment improved behavioral changes induced by CUMS. These findings demonstrate that NAMPT-mediated NAD synthesis responds to depression.

Thus, we prioritize three abnormalities that have proven the most effective in preclinical models [26]: anhedonia, cognitive impairment, and despair-like behavior. Because of their complexity, depression-related symptoms are challenging to evaluate through only one animal behavioral test. The FST and TST are reportedly used to evaluate depression in rodents, as evidenced by decreased or increased immobility time. These effects were induced by NAMPT knockout in the PFC and reversed by NR treatment. However, they could also be affected by general activity levels. As anhedonia is the primary symptom of depression, the SPT was used to investigate depression-like behavior in mice. The *Nampt*^flox/flox^ mice showed reduced sucrose preference ratios, which was similar to rats with CUMS-induced depression. Nevertheless, no difference in liquid intake was observed between groups, which indicated that the decrease in sucrose preference ratio did not depend on the decrease in total liquid consumption. All results verified that the depression model adopted in this study was effectively developed with CUMS-induced rats and *Nampt*^flox/flox^ mice. The NR changed these actions.

In addition to SPT, FST, and TST, OFT, NORT, SIT, and DTT could be used to evaluate behaviors in animals with depression, reflecting locomotor activity [27], cognitive function [13], social active ability, and social dominant position [15], respectively. Our study showed results which were consistent with those of previous reports. NR exerts protective effects against depression.

Previous studies have demonstrated that NAD content affects the level or activity of BDNF, SIRT1, AKT, and CREB, thereby affecting their biological effects. Chang et al. concluded that low NAD levels led to decreased activity-dependent BDNF expression [28]. Treatment with NMN or NAD ameliorated the degeneration of corneal nerve fibers by increasing the activation of SIRT1, CREB, and AKT. This finding is consistent with the results of our previous study. BDNF, the most important neurotropin in the brain, is a candidate target for the treatment of depression. BDNF activates various pathways including CREB, and CREB phosphorylation promotes BDNF transcription. This has been exploited by many antidepressants. Based on our results, it is speculated that NAMPT-NAD is a likely target for increasing BDNF-CREB expression in the PFC and hippocampus and exerts antidepressant effects.

Meanwhile, changes in CORT and monoamine neurotransmitters revealed that *Nampt*^flox/flox^ and NR both had an impact on the activity of the HPA axis. This was consistent with the results reported by Li et al. [29]. The HPA axis is also an action target of classic clinical antidepressants.

In conclusion, NR, a precursor of NAD, may boost NAD levels and improve the downbeat mood, and the inhibition of NAMPT in the PFC can deteriorate depression-associated behavior and cognitive function in mice. These results lay the groundwork for further investigation of the underlying mechanisms to identify targets to treat depression. It also demonstrates the critical involvement of NAMPT-mediated NAD synthesis in depression.

## Data Availability

The data used to support the findings of this study have been included in this article. The raw data were deposited at the following link: https://www.jianguoyun.com/p/DVTbJhsQioD3ChjQv9cEIAA (accessed on 11 October 2022).
